# Charge Redistribution Mitigation and Optimization for Sensor–ADC Interfacing in Low Cost Embedded Applications

**DOI:** 10.3390/s25164960

**Published:** 2025-08-11

**Authors:** Boyan Shabanski, Angel Marinov

**Affiliations:** Department of Electronics and Microelectronics, Faculty of Computer Sciences and Automation, Technical University of Varna, 9010 Varna, Bulgaria; bshabanski@tu-varna.bg

**Keywords:** ADC interface, capacitive precharging, charge redistribution, multiplexer, embedded systems, transient effects, Gray code, charge injection, sensor accuracy, low-cost design

## Abstract

This paper proposes a cost-effective five-component discrete capacitive pre-charging circuit designed to mitigate charge redistribution effects in Analog-to-Digital Converter (ADC) inputs, particularly for low-cost embedded applications involving multiplexed high-output-resistance sources. The paper presents an analysis and experimental validation of this approach, comparing its performance against traditional methods like grounding or leaving unused multiplexer inputs floating. The proposed solution leverages external components (two capacitors and three switches) and multiplexer features to pre-charge ADC inputs to approximately half the reference voltage, which could be taken directly from the multiplexer supply rail, significantly reducing transient glitches and settling times. The experimental results demonstrate a clear improvement, achieving settling times up to 1.4 µs shorter than conventional approaches during specific multiplexer transitions. Component selection guidelines are outlined, including compensation capacitor sizing and transistor choice, addressing practical concerns such as charge injection effects. Despite certain experimental constraints noted during testing, the developed discrete pre-charging method consistently exhibited substantial performance gains. Our findings confirm that this practical, minimal-component strategy effectively addresses charge redistribution challenges, presenting an efficient solution for enhancing ADC input accuracy and response speed in resource-limited embedded sensor systems.

## 1. Introduction

Analog-to-Digital Converters (ADCs) represent critical building blocks in modern embedded sensor applications, providing an essential interface between analog signals and digital processing units. Accurate signal acquisition and conversion are essential, particularly in systems that involve multiplexed signals from high-output-resistance sensor sources. Such scenarios commonly arise in low-cost embedded solutions and resource-constrained sensor nodes, including those powered by energy harvesting, deployed in wireless sensor networks, and integrated within Internet-of-Things (IoT) systems [1,2,3,4,5].

However, interfacing high-impedance sensor sources with multiplexed ADC inputs often results in detrimental transient effects, primarily caused by charge redistribution at the ADC inputs. These transient effects manifest as undesired voltage spikes, transient glitches, and prolonged settling times, significantly limiting the accuracy and reliability of sensor readings. In practical applications, these transient responses directly affect the system’s performance by increasing measurement uncertainty, decreasing effective resolution, and elevating overall power consumption due to extended sampling durations [6,7,8,9,10,11,12,13,14,15,16,17,18,19,20].

Several well-established approaches are available for managing multiplexed ADC input transients. Common recommendations from semiconductor manufacturers include grounding unused multiplexer inputs or leaving them floating [21,22,23,24,25,26,27,28,29,30,31,32]. Although straightforward, these methods have inherent drawbacks. Grounding unused inputs causes repetitive charging cycles, extending the required settling period, whereas floating inputs introduce unpredictable transient responses. Advanced integrated ADC solutions often mitigate such problems internally through pre-charging or buffered inputs, but such built-in features are typically absent in cost-sensitive, low-power microcontrollers [21,22,23,24,25,26,27,28,29,30,31,32,33,34,35,36,37].

Not adequately addressing these transient and charge redistribution effects has severe practical implications. Systems may suffer from increased measurement inaccuracies, heightened power consumption due to prolonged ADC sampling periods, and reduced lifetime in battery-operated and energy-harvesting sensor applications. Specifically, unreliable analog measurements can propagate erroneous data into downstream processing, impairing the reliability of decision-making processes and analytics based on sensor data [38,39,40,41].

To address these challenges, discrete external capacitive pre-charging circuits have been explored and were shown to be effective. Previous studies have shown that these pre-charging circuits significantly reduce transient response issues, stabilize ADC inputs, and improve overall measurement accuracy [38,39,40,41]. Furthermore, digital hazard mitigation techniques such as Gray code have been recommended to minimize switching transients and ensure stable multiplexer transitions [42]. Similarly, addressing charge injection issues via carefully selected transistors, dummy switches, and matched transistor pairs further enhances ADC performance and reliability [33,34,35,36,37].

The current paper builds upon these foundations and proposes a discrete capacitive pre-charging circuit specifically optimized for low-cost embedded ADC interfacing applications. The approach integrates minimal external components alongside readily available multiplexer features to mitigate charge redistribution effectively. This study experimentally validates the proposed solution against conventional practices (grounding and floating), highlighting significant improvements in transient performance and ADC accuracy. In addition, this work details practical guidelines for selecting compensation capacitors and switching transistors to address charge injection and transient effects comprehensively.

This proposed discrete capacitive pre-charging circuit thus represents a practical solution directly relevant to sensor systems powered by energy-harvesting technologies, contributing to improved measurement accuracy, enhanced reliability, and lower power consumption in IoT and wireless sensor applications [1,2,3,4,5].

## 2. Materials and Methods

### 2.1. Circuit Overview and Principle of Operation

This work proposes a discrete capacitive pre-charging circuit designed to mitigate charge redistribution transients at ADC (Analog-to-Digital Converter) inputs in low-cost embedded systems. These transients commonly arise when switching between high-output-impedance sources via analog multiplexers, where abrupt changes in the input node voltage result in long settling times and measurement inaccuracies. Such problems are particularly pronounced in battery-powered and energy-sensitive applications, including wireless sensor nodes and energy-harvesting devices, where minimizing conversion latency is critical for power efficiency and overall throughput.

To address this, the circuit uses a pre-charging method in which the ADC input is actively conditioned before being reconnected to a new signal source. This is achieved by isolating the input node during transitions and charging it to an intermediate voltage—approximately half of the reference voltage line (VREF/2)—via a compensation capacitor. Pre-charging to this midpoint reduces the voltage difference between successive sources and the ADC’s internal sampling capacitor, minimizing the required recovery time and power dissipation. The pre-charging scheme reference voltage is intended to be an already available supply source, thus removing the need for a dedicated reference source while avoiding additional loading to the ADC internal reference source.

The experimental implementation is based on the 74HC4851 multiplexer (Texas Instruments Incorporated, Dallas, TX, USA; purchased from Mouser Electronics, Mansfield, TX, USA), a widely available, low-cost 8:1 analog switch with a controllable output enable (EN_N) line. This line allows the output to be electrically disconnected from all input channels during transitions. The pre-charging sequence is controlled via GPIO (General Purpose Input/Output) lines from an STM32 microcontroller (For this paper STM32F411RE, STMicroelectronics, Geneva, Switzerland; purchased from Mouser Electronics, Mansfield, TX, USA). STM32 was selected for its flexible I/O architecture, precise timer peripherals, and atomic bit-level control using the Bit Set/Reset Register (BSRR), all of which support safe and deterministic switching behavior.

While STM32 was chosen for demonstration, the proposed approach is not platform-specific. Any microcontroller with digital I/O and timer support—including AVR, PIC, or MSP430 families—can implement the control sequence, with minor adaptation to timing and pin configuration. Similarly, although 74HC4851 was used here, the method is fully applicable to other multiplexers such as 74LV4051 or ADG series, depending on voltage range, speed, and footprint needs. For more complex systems, CPLDs (Complex Programmable Logic Devices) or FPGAs (Field Programmable Gate Arrays) could integrate the entire switching and control logic in a reconfigurable digital block

The circuit architecture centers on three external switches—two PMOS (SW_A, SW_B) and one NMOS (SW_C)—coordinated in a three-phase sequence:

- Hi-Z (Idle): All switches are off, and the ADC node is isolated.
- Reset Phase: The node is pulled to ground through SW_C to ensure a known low level.
- Pre-Charge Phase: The node is charged toward VREF/2 via SW_B and CCOMP.

**Figure 1:**
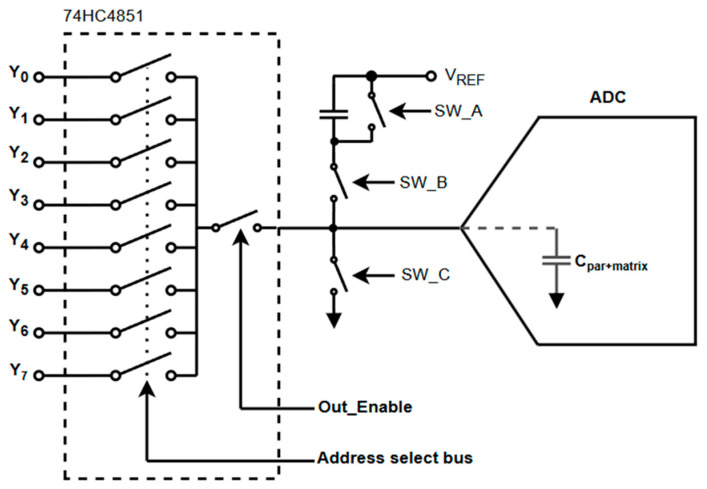
Proposed topology for capacitive pre-charging of ADC input.

**Figure 2:**
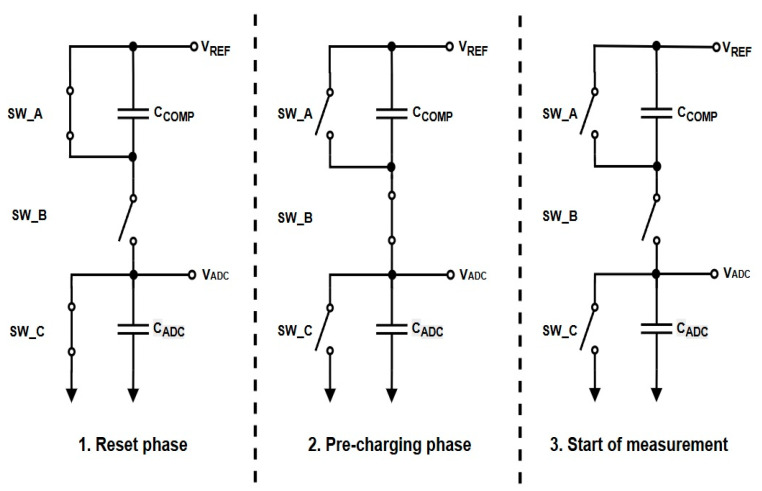
Proposed pre-charging circuit operating phases.

This switching logic is illustrated in the topology diagram (Figure 1), and the control timing is shown on Figure 2. The coordination between the EN_N pin and the external switches enables clean isolation and staged reconnection of the ADC input, minimizing charge injection and transient artifacts.

The use of low-cost discrete components and software-controlled timing makes this method accessible and practical for a wide range of embedded systems. With only a few passive and active components, it provides a measurable improvement in signal conditioning without the need for op-amps, dedicated reference voltage buffers, or high-speed switching logic.

### 2.2. Control Logic and Switching Operation

The functionality of the discrete pre-charging circuit is enabled by a precisely timed control sequence that governs the behavior of the external switches (SW_A, SW_B, SW_C) and the multiplexer′s output enable line (EN_N). This sequence is executed by a microcontroller via digital I/O lines and must follow a strict non-overlapping pattern to avoid direct conduction paths between VREF and GND. Timing errors or improper sequencing can lead to unwanted short circuits, residual voltage errors, or increased power consumption.

To ensure robust operation, the control logic is implemented as a four-phase state machine consisting of the following stages:

- Hi-Z (Idle): All switches are off; the ADC input is electrically isolated.
- Reset Phase: The ADC input is pulled to ground through SW_C, discharging any residual charge.
- Break-Before-Make (BBM): A brief delay is introduced to ensure that no two switches are active simultaneously.
- Pre-Charge Phase: The node is charged toward half of the reference voltage line (VREF/2) through SW_B and the compensation capacitor.

The GPIO outputs (General Purpose Input/Output) for each phase are programmed using STM32′s BSRR (Bit Set/Reset Register), which enables atomic updates to all control lines. This prevents glitches caused by partial state transitions, particularly important when switching multiple pins simultaneously. Table 1 summarizes the control line states, register-level bitmask equivalents, and phase durations.

Each phase duration was initially estimated based on RC constants derived from the transistor switching speed and ADC node capacitance, then empirically adjusted for stable performance under varying input conditions. The Break-Before-Make interval ensures that SW_C is fully deactivated before SW_B is turned on, preventing momentary shoot-through between the supply rails.

**Figure 3:**
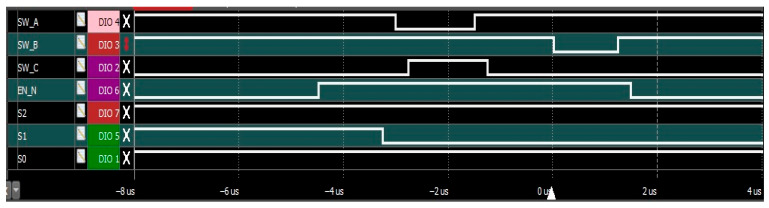
Captured waveform for the control lines on the experimental setup at a low switching frequency.

Control waveforms for the full sequence are shown in Figure 3. These were captured under real-time operation and demonstrate clean signal transitions, well-defined timing, and the absence of switching artifacts. Transitions between logic states are synchronized with multiplexer address updates to maintain signal integrity across switching events.

While the STM32 microcontroller was used in this implementation due to its built-in BSRR support and high-resolution timers, the control logic is generalizable to other MCU families. On AVR platforms, for example, port-level writes can accomplish similar atomic updates. Timing precision may vary depending on clock speed and peripheral capabilities, but the overall logic structure remains consistent across platforms.

This discrete, software-driven control scheme provides a flexible and resource-efficient method for implementing safe, deterministic switching in low-cost analog front-ends. Its compatibility with general purpose microcontrollers and minimal hardware overhead make it attractive for sensor interface applications where ADC performance must be maintained without complex analog signal conditioning.

### 2.3. Component Selection and Charge Injection Mitigation

The performance of the discrete pre-charging circuit depends not only on its control logic but also on the characteristics of the switching components. In particular, careful selection of the external transistors is essential for minimizing charge injection, matching impedance across discharge paths, and achieving consistent transient response. Poorly matched or suboptimal devices can introduce voltage offsets, non-linear settling behavior, or timing inconsistencies that degrade ADC accuracy.

The circuit uses two PMOS transistors (SW_A and SW_B) and one NMOS transistor (SW_C) in accordance with Figure 1 and Figure 2. During the reset phase, SW_C discharges the ADC input node to ground, while SW_A may act as an auxiliary control element depending on the layout. SW_B is responsible for sourcing charge from the compensation capacitor to the ADC input during the pre-charge phase. For proper operation, SW_A and SW_C—when used in combination—should present a symmetrical discharge path, minimizing imbalances that could result in residual voltage artifacts.

**Figure 4:**
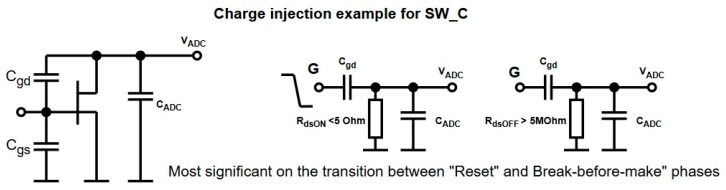
Charge injection example and equivalent circuit for SW_C in reset/BBM phases.

One of the main challenges in this configuration is charge injection, particularly during fast switching of SW_C. When the NMOS gate transitions from HIGH to LOW, the abrupt change in gate voltage couples charge through C_GD_ into the ADC input capacitance, resulting in a transient voltage step on the ADC node (Figure 4).

Voltage at V_ADC_ at the end of the BBM phase could be approximated by omitting the effect of the decaying MOSFET channel following the series capacitor charge distribution formula:

(1) $$V_{ADC\_BBM}\approx -\frac{C_{GD}{(V}_{GS}-V_{th})\;+\;C_{ADC}V_{RESET}}{C_{GD}\;+\;C_{ADC}}$$

where *C_GD_* is SW_C gate-drain capacitance, *V_GS_* is the applied voltage at the gate (since *C_GD_* “inner” plate is shorted to GND via the MOSFET channel), and *V_RESET_* is the voltage on *C_ADC_* at the end of the reset phase. The minus sign reflects *V_ADC_* becoming lower than GND as a result of the charge injection. The above approximation is based on the formula used for estimating the charge injection effect for transistors at an integrated level, which proposes differentiating between two cases, slow rise/fall times and fast rise fall times, for better estimation accuracy [26].

**Figure 5:**
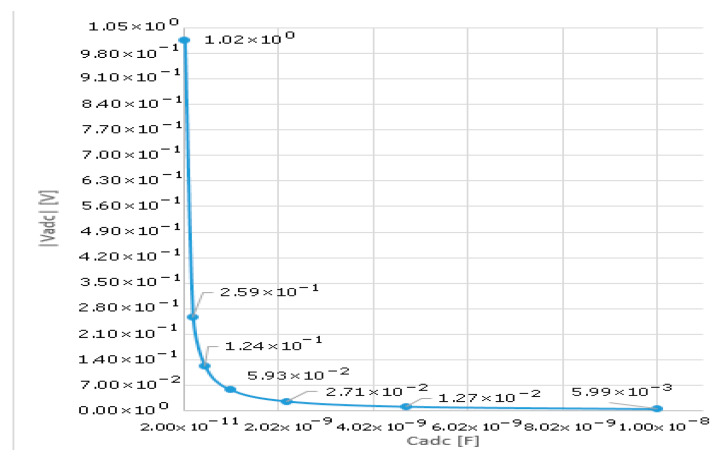
Charge injection related undershoot in reset phase for SW_C—BS170, Vgs = 5 V versus different values of CADC.

Substituting in (1) for the transistor used as SW_C (BS170) with gate-drain assumed to be half of the C_ISS_ listed per datasheet (24 to 40 pF), the following graph could be plotted (Figure 5) for a rough estimate of the impact of charge injection versus various values of C_ADC_.

For the purposes of the experiment, a starting value of 100 nF was chosen for C_COMP_ and C_ADC_, mimicking the ADC input matrix, which makes charge injection effects negligible. This however comes with the added drawback of increasing the settling time required for the signal source to drive from Vref/2 to its final value.

The overall error for the pre-charging scheme due to charge injection leads to offset from its desired set point of Vref/2; thus, Vadc at the end of the pre-charging phase would approximate:

(2) $$V_{ADC\_prech}\approx \frac{C_{ADC}\left(\frac{V_{REF}}{2}-V_{Inj_{C}}-V_{inj_{B}}\right)+\;C_{Comp}(\frac{V_{REF}}{2}-V_{InjB}+V_{InjA})}{C_{GD}\;+\;C_{ADC}}$$

where *V_InjA_*, *V_InjB_*, and *V_InjC_* are the voltage error due to charge injection from switches A, B, and C, respectively, calculated as per Equation (1).

**Figure 6:**
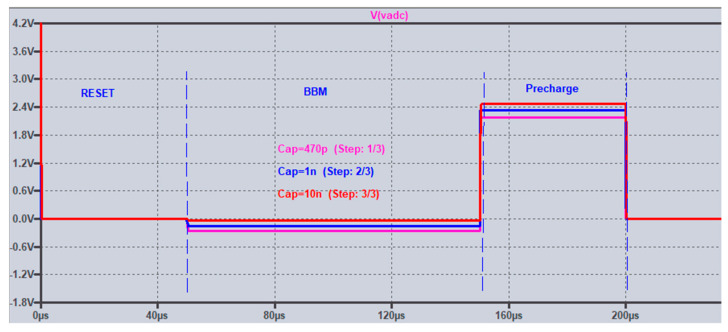
LTspice parametric simulation for charge injection related undershoot in reset phase for SW_C versus different values of C_ADC_.

A parametric simulation for this behavior was setup with 3 values of C_COMP_ and C_ADC_ (470 pF, 1 nF, and 10 nF). The related LTspice file can be found on github [43]. The result is shown in Figure 6.

To mitigate these effects, several design considerations were implemented:

- Low gate charge transistors were selected to reduce the amount of injected charge. Devices with low *Q_G_* and minimal *C_GS_* and *C_GD_* were prioritized.
- Matched transistor pairs were evaluated to ensure symmetrical impedance and switching behavior. This is particularly useful for implementing dummy switches or balancing discharge and charge paths.
- Dynamic voltage scaling could be applied to the gate control signals where possible, reducing the amplitude of the control voltage swing and hence the energy coupled during switching transitions [14,15,20].
- Component packaging was considered: dual-matched transistor packages such as 2N7002AKRA-QZ (dual NMOS, Diodes Incorporated, Plano, TX, USA; purchased from Mouser Electronics, Mansfield, TX, USA) offered good matching characteristics and compact layout benefits.

**Figure 7:**
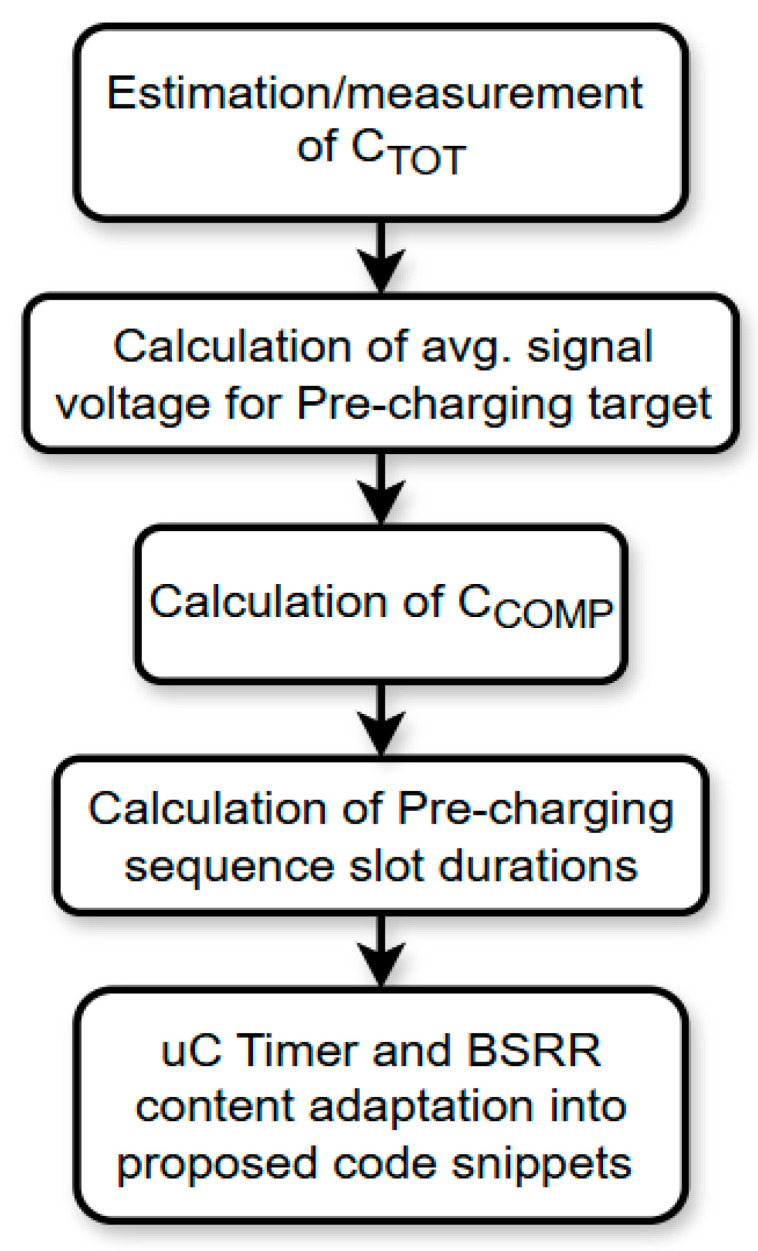
Flow diagram of pre-charging scheme component and parameter selection.

The component selection process is summarized in Figure 7, which outlines the decision flow based on key transistor parameters. RDSON (MOSFET ON Resistance) values were evaluated under operating voltage conditions to ensure that the discharge and pre-charge paths provided sufficiently low impedance without drawing excessive current or introducing signal distortion.

In addition to electrical properties, layout considerations played a significant role in minimizing charge injection and crosstalk. Short trace lengths, local decoupling, and grounded guard traces were used to contain transient currents and reduce unintended coupling into sensitive nodes.

The combined effect of these mitigation strategies is a well-controlled pre-charging profile with minimal overshoot, reproducible voltage levels, and improved transition consistency across a variety of input conditions. By carefully selecting and applying discrete transistors in this topology, the circuit avoids the complexity of analog buffers while still achieving stable and repeatable input conditioning suitable for high-resolution ADC applications.

### 2.4. Compensation Capacitor Sizing and Parasitic Estimation

The sizing of the compensation capacitor ($C_{COMP}$) is a critical design element in the proposed pre-charging system. Its role is to establish an intermediate voltage at the ADC input during the pre-charge phase, thereby reducing the voltage step that would otherwise occur during multiplexer transitions. Properly sizing $C_{COMP}$ ensures that this intermediate level approaches $V_{REF}/2$, minimizing the settling time required for subsequent ADC sampling.

To determine an appropriate value for $C_{COMP}$, the charge redistribution principle is used. The final voltage ($V_{final}$) achieved by pre-charging can be modeled using capacitive charge sharing between $C_{COMP}$ and the effective input capacitance seen at the ADC node. This includes contributions from the ADC itself, the multiplexer output, and PCB parasitics. The capacitor sizing is thus approximated using the following:

(3) $$C_{COMP}\approx C_{ADC}\;+\;C_{MUX}\;+\;C_{TRACE}$$

where $C_{COMP}$ is the compensation capacitor to be selected, $C_{ADC}$ is the input capacitance of the ADC (typically 5–20 pF), $C_{MUX}$ is the output capacitance of the multiplexer, and $C_{TRACE}$ represents parasitic capacitance from Printed Circuit Board (PCB) routing and pads.

#### 2.4.1. Analytical Estimation

Initial sizing of $C_{TRACE}$ was performed using the Saturn PCB Toolkit [44], which estimates PCB trace parasitics based on trace geometry and substrate properties. For standard 2-layer FR-4 PCB with 50 mm trace length and 0.3 mm width, $C_{TRACE}$ was estimated at 25–30 pF. Including nominal values for $C_{ADC}$ and $C_{MUX}$, the total effective capacitance at the ADC input node was estimated to fall between 60 and 80 pF.

#### 2.4.2. Empirical Measurement

**Figure 8:**
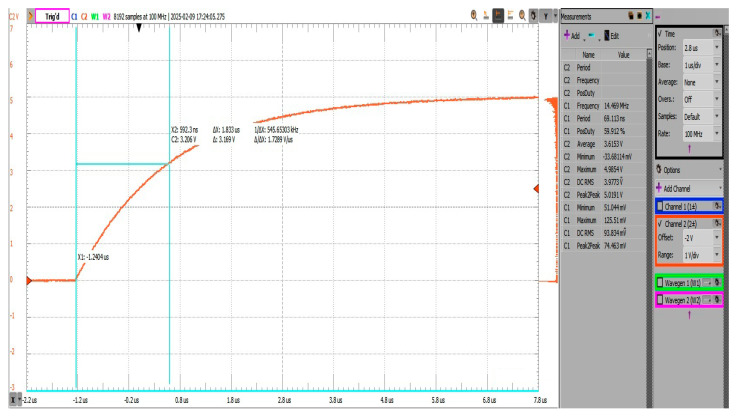
Estimation of trace capacitance by injecting square wave through known resistor.

To validate this estimate, a time constant τ measurement was carried out using a resistor–capacitor test setup. A known resistor R=11kΩ was placed in series with the ADC node, and a square waveform generated with the built-in generator of an “Analog Discovery” device was applied. The time for the node voltage to reach 63.2% of its final value was measured as the circuit’s time constant τ (Figure 8). Using the following relationship:

(4) $$\tau \approx R.C\to C\;=\;\frac{\tau }{R}$$

where τ is the RC time constant (in seconds), *R* is the series resistor used, and *C* is the total capacitance at the ADC node.

Substituting the measured value.

(5) $$\tau =\frac{2.058\;\mu s}{11\;k\Omega }\approx 187\;pF$$

After accounting for the capacitance of the oscilloscope probe UT-P03, which is around ~100 pF in × 1 configuration, the actual system capacitance was inferred to be approximately 87 pF. The board was configured such that DIP Switch U9 had only connection 1 to 6 closed, connecting only the mux U1 to the output node. Control lines for “*EN_N*”, *“SW_A”*, and *“SW_B”* were set to a “logic HIGH”, so the line was not actively driven by sources other than the injection resistor of 11 kΩ via the signal generator. The oscilloscope probe was placed at the injection point past the resistor.

### 2.5. Firmware Implementation and Digital Control

As mentioned in Section 2.1, the pre-charging sequence is implemented on an STM32 microcontroller. For development convenience, the STM32 Nucleo-F411RE board (STMicroelectronics, Geneva, Switzerland; purchased from Mouser Electronics, Mansfield, TX, USA) was used, providing access to precise timer peripherals and the Bit Set/Reset Register (BSRR), which enables atomic GPIO updates without timing glitches.

The firmware operates as a non-blocking timer-driven state machine that cycles through the four control phases—Hi-Z (Idle/High-Impedance state), reset, Break-Before-Make (BBM), and pre-charge. Each phase is represented by a predefined GPIO bitmask and a timer reload value. A hardware timer manages the transitions, ensuring consistent timing resolution and phase sequencing. The core control logic is as follows:

currentStateIndex = (currentStateIndex + 1) % 4;

GPIOs (General Purpose Input/Output) for the switches and multiplexer enable line are assigned to a single port to ensure synchronized updates. The durations for each phase were calibrated based on measured RC constants (see Section 2.4) and verified through oscilloscope observation. This timing structure allows the pre-charge and discharge phases to be completed reliably within a microsecond-scale control loop.

The firmware also manages multiplexer address sequencing using Gray code to minimize simultaneous bit transitions, reducing the risk of digital glitches coupling into the analog domain.

The configuration templates, related code routines for STM32 controller/Arduino, and all associated design files (full schematic, Printed Circuit Board (PCB), Bill of Materials (BOM), and others) are available on github [43].

This implementation demonstrates that accurate and flexible analog input conditioning can be achieved through structured firmware, with minimal hardware requirements and broad portability across embedded platforms.

## 3. Experimental Validation and Discussion

**Figure 9:**
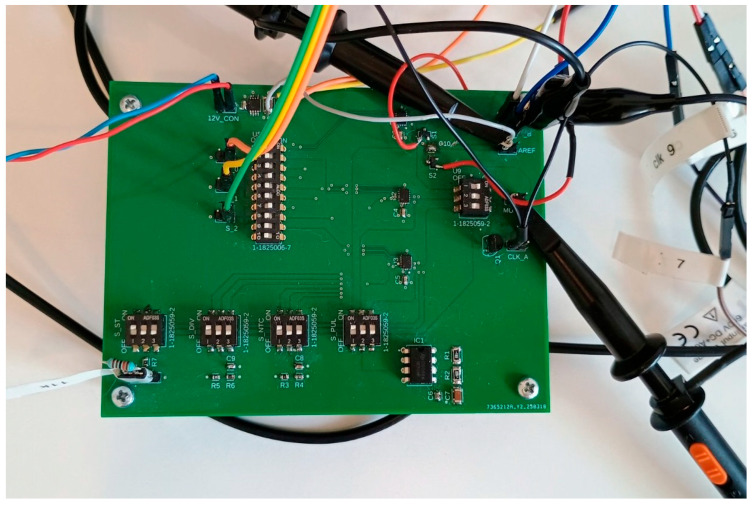
Experimental setup of PCB.

To validate the effectiveness of the proposed discrete capacitive pre-charging circuit, it was implemented and tested alongside two widely used baseline configurations: grounded and floating multiplexer inputs. The evaluation focused on characterizing the transient behavior of the ADC input during channel-switching events, with an emphasis on measuring settling time as the key performance metric. Each configuration was realized on the same test platform under identical conditions (Figure 9), and waveform data was collected to compare how quickly and consistently the ADC (Analog-to-Digital Converter) input stabilized after a multiplexer address change. By analyzing both moderate and worst case switching scenarios, we demonstrate how the pre-charging method reduces charge redistribution effects, accelerates input settling, and improves the timing efficiency of multiplexed sensor systems.

### 3.1. Experimental Configurations

**Figure 10:**
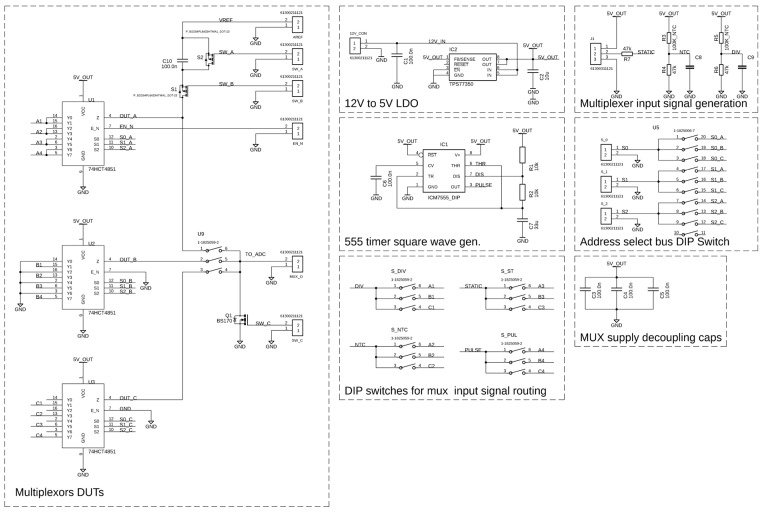
Complete schematic of used experimental board.

To evaluate the effectiveness of different multiplexer input-handling strategies, three circuit configurations were implemented and tested under controlled conditions. The complete experimental setup, including the ADC, multiplexer, pre-charging circuitry, and associated control logic, is shown in Figure 10. Each configuration was assessed for its influence on input node settling behavior, charge injection, and overall measurement stability, providing a basis for the comparative results presented in the following subsections.

#### 3.1.1. Grounded Unused Inputs—Conventional Approach 1

**Figure 11:**
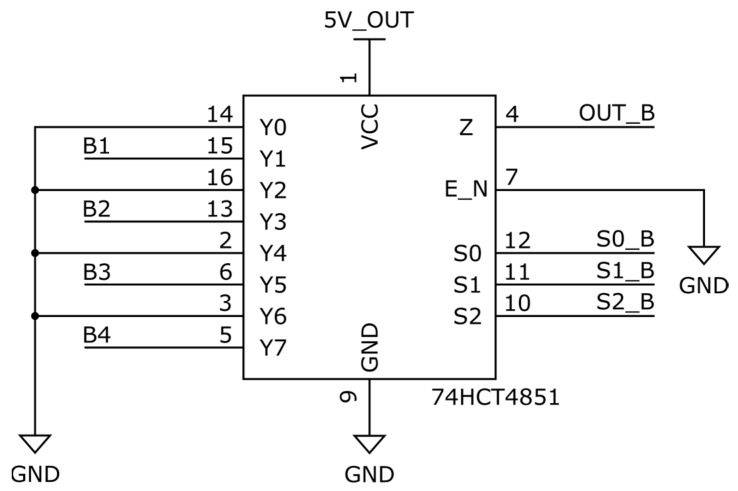
MUX with grounded unused inputs.

This configuration (Figure 11) follows the standard recommendation of tying all unused multiplexer inputs to ground potential. In this case, the active channel is selected while all others remain at 0 V, forcing the multiplexer output to reset to a defined low level during each transition. As a result, the ADC input node begins each sampling event from zero, regardless of the previous input’s voltage. Although this guarantees a predictable baseline and eliminates any risk of floating voltages, it also means that each new sensor input must fully charge the ADC node from 0 V to its final value. This presents a significant disadvantage when interfacing with high-output-impedance sensors or low-bandwidth sources, where the charging time constant becomes a bottleneck. The result is longer settling times after each switch, reduced measurement speed, and potential sampling errors if conversion begins prematurely.

#### 3.1.2. Floating Unused Inputs—Conventional Approach 2

**Figure 12:**
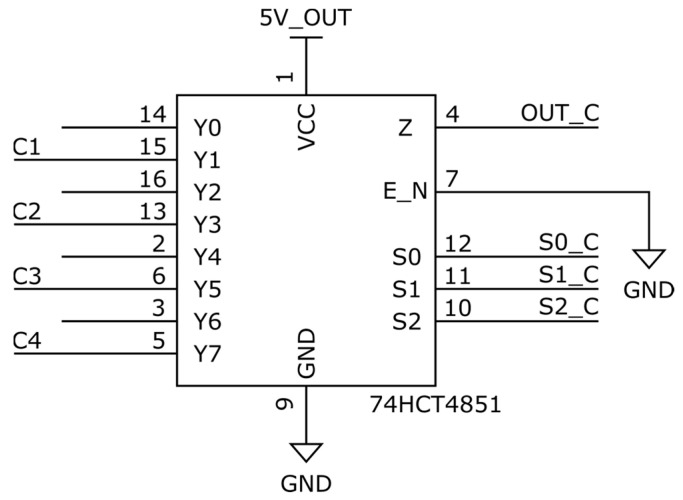
MUX with floating unused inputs.

In this setup (Figure 12), the unused channels on the multiplexer are left unconnected (floating), and the channel address is cycled through a deliberately inserted “dummy” channel before selecting the next active sensor. This strategy increases the effective time between consecutive sensor measurements, allowing the ADC node to naturally discharge or equilibrate before the next connection is made. While the intention is to reduce transient overshoot or charge injection by allowing more time for passive settling, the actual voltage at the ADC input becomes unpredictable, depending on leakage currents, parasitic coupling, and the previous channel’s voltage level. Additionally, since only every other multiplexer cycle yields a valid measurement, the effective throughput is halved. This approach may reduce immediate transients but does not provide controlled or consistent settling behavior, especially in dynamic or noisy environments.

#### 3.1.3. Capacitive Pre-Charging Scheme—Proposed Approach

**Figure 13:**
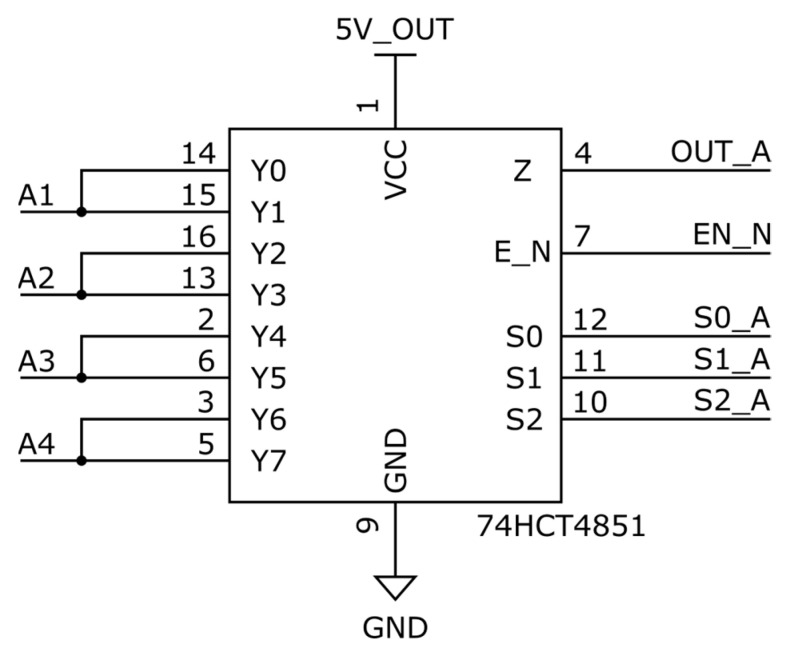
MUX for pre-charging scheme evaluation.

The third configuration (Figure 13) introduces a discrete pre-charging circuit designed to actively condition the ADC input node before each new sensor input is selected. The pre-charging mechanism uses three MOSFET switches (SW_A, SW_B, SW_C) and a compensation capacitor (CCOMP), driven by firmware-controlled GPIO logic. During the transition phase, the multiplexer output is disconnected using the 74HC4851′s EN_N line, and the node is charged to an intermediate level, approximately VREF/2. This ensures that the voltage difference between the node and the next sensor input is minimized, thereby reducing the magnitude of charge redistribution required. Additionally, the multiplexer address lines are sequenced using a Gray code scheme to limit the number of simultaneous switching bits and reduce digital noise coupling. Timing control is precisely managed using microcontroller hardware timers and atomic port manipulation (BSRR), ensuring reliable, repeatable behavior. This method introduces minimal hardware overhead and no significant energy penalty but offers substantial improvements in settling time and measurement repeatability, particularly in high-impedance or low-power applications.

### 3.2. Transient Response and Settling Time Comparison

To quantify the performance differences among the three configurations, we captured and analyzed the ADC input waveforms during specific channel-switching events. Two representative cases were selected:

- Moderate voltage step: Switching from address 1 to 3 (binary 001 → 011).
- Large voltage step: Switching from address 7 to 5 (binary 111 → 101).

These transitions represent typical and worst case scenarios, respectively, in terms of voltage difference and required charge redistribution. In each case, settling time was defined as the time needed for the ADC input to reach either 10–90% of its final value (for moderate steps) or within ±10% of the final steady state voltage (for large steps).

#### 3.2.1. Moderate Step: Address 1 → 3

**Figure 14:**
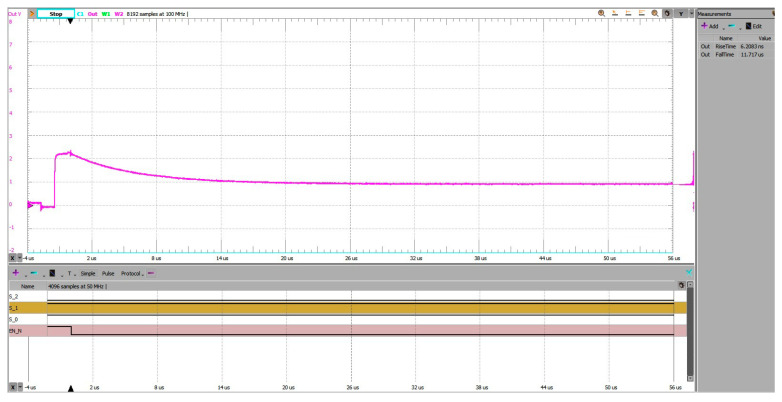
Address change 1 to 3, experimental setup 3 (pre-charging). Settling time, 10–90%, is 11.717 µs on falling edge.

Figure 14 shows the transient waveform captured when using the proposed pre-charging scheme. The ADC input was pre-charged to an intermediate voltage before switching to channel 3, resulting in a smooth transition with minimal overshoot. The measured 10–90% settling time was approximately 11.7 µs.

**Figure 15:**
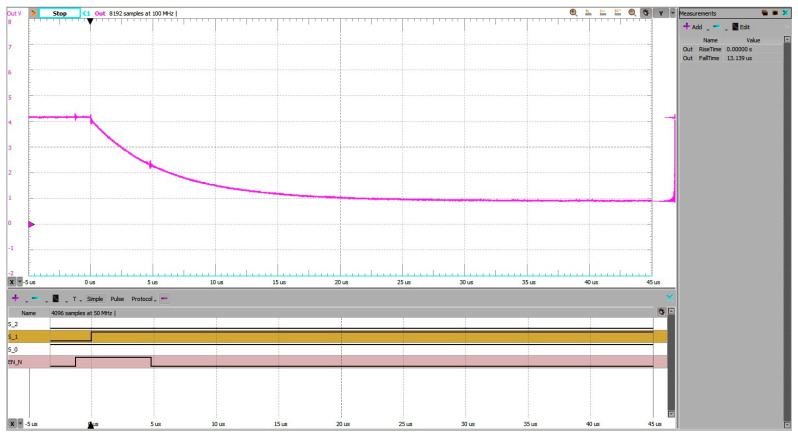
Address change 1 to 3, experimental setup 1 (grounded unused inputs). Settling time, 10–90%, is 13.139µs on falling edge.

Figure 15 shows the corresponding waveform for the grounded-input method. Starting from 0 V, the ADC input had to charge fully to the target voltage. This produced a longer transition with more pronounced overshoot and a 10–90% settling time of about 13.1 µs.

The difference—approximately 1.4 µs, or a 10–12% improvement—demonstrates the benefit of pre-charging in reducing the required settling interval, especially for high-impedance sources.

The floating-input approach was not explicitly plotted but would not substantially reduce settling time. While inserting a dummy (floating) channel between address transitions might passively reduce the initial voltage jump, it would require an extra sampling cycle and offer no active control of the ADC input level.

#### 3.2.2. Large Step: Address 7 → 5

**Figure 16:**
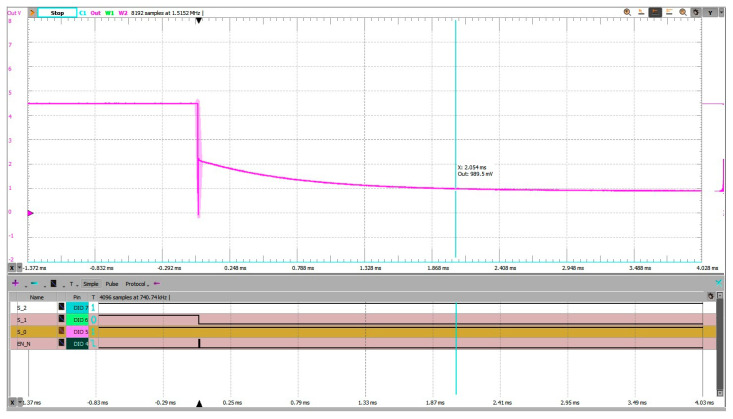
Address change 7 to 5, experimental setup 3 (pre-charging). Settling time measured from trigger on falling edge on EN_N to within 10% of steady state voltage is 2.054 ms.

In Figure 16, the pre-charging scheme reduced the settling time to approximately 2.05 ms. The ADC input, starting near half of the reference voltage line (VREF/2), exhibited a moderated transient and a controlled exponential decay toward the final voltage.

**Figure 17:**
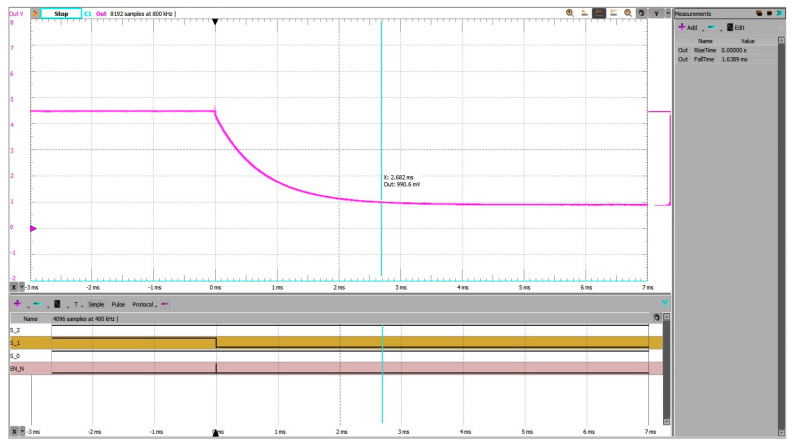
Address change 7 to 5, experimental setup 1 (grounded unused inputs). Settling time measured from trigger on falling edge on EN_N to within 10% of steady state voltage is 2.682 ms.

In Figure 17, the grounded-input method resulted in a significantly larger transient and a settling time of approximately 2.68 ms. The source had to drive the node from 0 V to the final voltage through high-impedance paths, resulting in slower convergence.

The pre-charging method thus yielded a reduction of 0.63 ms, or about 23% faster settling, compared to the grounded baseline.

As before, the floating-input method would add delay without active correction, effectively extending the measurement time without directly improving settling dynamics.

#### 3.2.3. Capacitor Sizing Effect on Charge Injection-Induced Offset Error

This section shows the experimental results for the charge injection-induces offset error during the RESET, Break-Before-Make (BBM), and Hi Z phases of operation. Behavior was captured for a fixed multiplexer address. Ccomp and Cadc values tested include 470 pF, 1 nF, 2.2 nF, 22 nF, and 47 nF.

**Figure 18:**
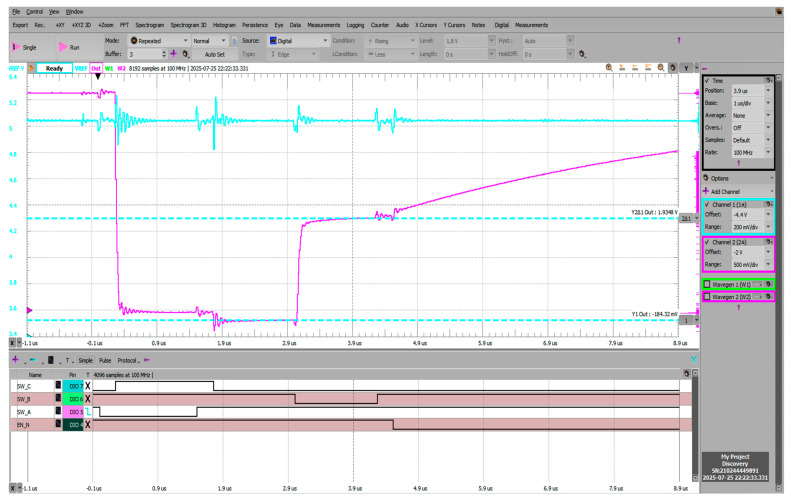
Transient behavior during reset-BBM-pre-charge phases; C*_COMP_* and C*_ADC_* = 470 pF.

Figure 18 shows the negative voltage offset during the reset phase visible at the Vadc node (pre-charging scheme output) reaching −184.32 mV, followed by the pre-charging phase where instead of the intended 2.5 V (Vref/2), voltage is settled at 1.9348 V. Pre-charge settling time was measured to be 70.18 ns, measured as 10–90% from the reset state (−184 mV) to the pre-charged voltage (1.934 V).

**Figure 19:**
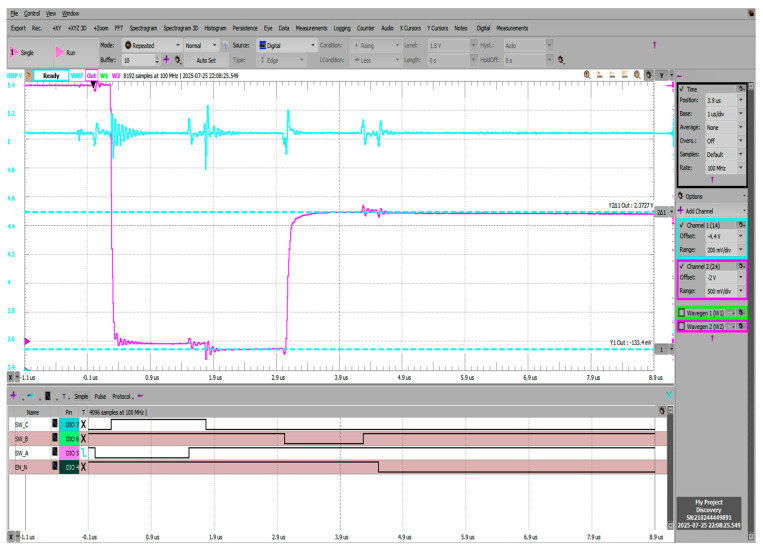
Transient behavior during reset-BBM-pre-charge phases; CCOMP and CADC = 1 nF.

Figure 19 shows the negative voltage offset during the reset phase visible at the Vadc node (pre-charging scheme output) reaching −133.4 mV, followed by the pre-charging phase where instead of the intended 2.5 V (Vref/2), voltage is settled at 2.372 V. Offset error is still considerable in both the reset and pre-charge phases. Pre-charge settling time was measured to be 77.67 ns, measured as 10–90% from the reset state (−133 mV) to the pre-charged voltage (2.372 V). The blue trace on channel 1 shows the Vref voltage and the ripple introduced by the pre-charging scheme operation. Worst case voltage dip magnitudes in this configuration reach 200 mV at the start of the reset phase.

**Figure 20:**
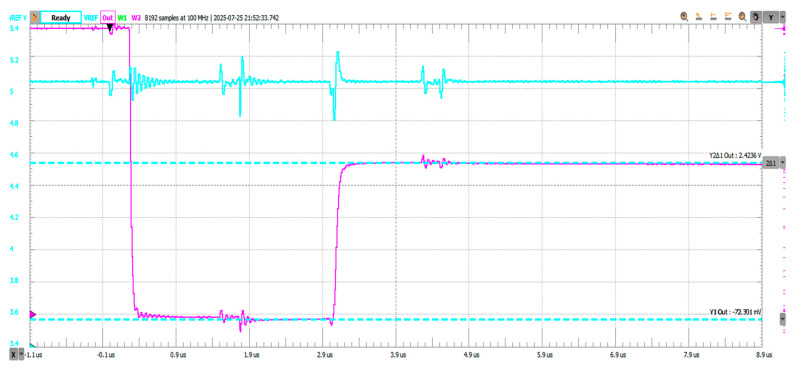
Transient behavior during reset-BBM-pre-charge phases; Ccomp and Cadc = 2.2 nF.

Figure 20 shows that the negative voltage offset during the reset phase reaches −72.3 mV at the Vadc node (pre-charging scheme output), followed by the pre-charging phase where the voltage settles at 2.423 V. Pre-charge settling time was measured to be 82.69 ns, measured as 10–90% from the reset state (−72.3 mV) to the pre-charged voltage (2.423 V). The blue trace on channel 1 shows the Vref voltage and the ripple introduced by the pre-charging scheme operation. Worst case voltage dip magnitudes in this configuration reach ~230 mV during the pre-charge phase, dictated by the reduction in Equivalent Series Resistance (ESR) associated with the larger value capacitors.

**Figure 21:**
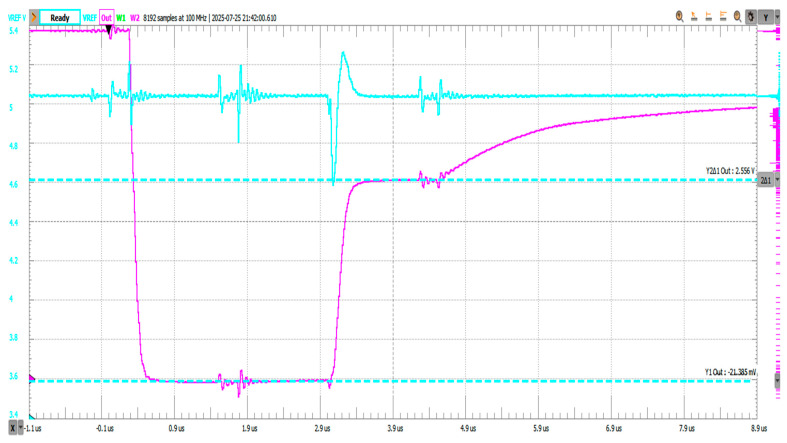
Transient behavior during reset-BBM-pre-charge phases; Ccomp and Cadc = 22 nF.

As is visible from Figure 21, charge injection is no longer an issue at such high Ccomp and Cadc values, as both the reset and pre-charge phases reach their expected voltage levels, near zero and near vref/2, respectively. However, the slope durations dictating the minimal duration of each phase have increased to near 200 ns, which reduces the overall sampling rate improvement of the proposed method.

Further increasing the used capacitor values does not bring any significant improvements in terms of charge injection. It does, however, highlight the transient loading on the reference voltage line (VREF), which is due to the decrease in ESR associated with the increased capacitance values. Worst case voltage dip magnitudes in this configuration reach ~430 mV during the pre-charge phase.

**Figure 22:**
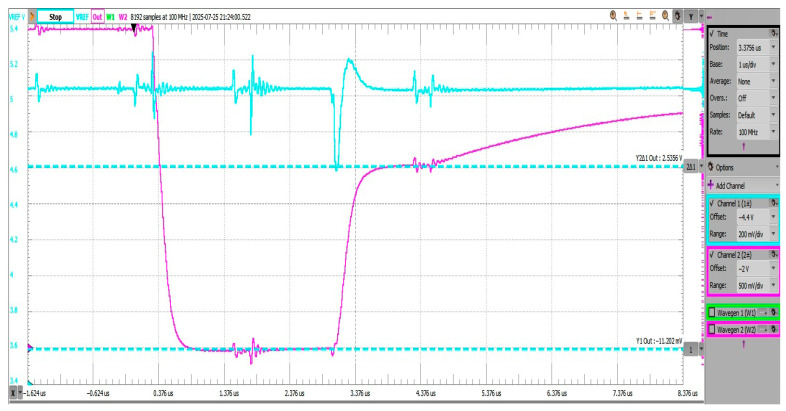
Transient behavior during reset-BBM-pre-charge phases; Ccomp and Cadc = 47 nF.

With both capacitors at a value of 47 nF, the charge injection undershoot and subsequent offset are no longer observable. Voltage at the reset phase was measured at around −11.2 mV, with a pre-charge voltage of 2.535 V. However, voltage dips in this configuration reach almost ~430 mV or nearly the same result as with the 22 nF configuration. This shows that voltage dip magnitude is becoming dominated by the RDSon of the pre-charge switch (SW_B) shown in Figure 22.

### 3.3. Performance Summary

The experimental data clearly demonstrate that the proposed discrete capacitive pre-charging scheme offers a consistent and measurable improvement in ADC input settling performance when compared to both conventional grounded and floating multiplexer input strategies.

- For a moderate voltage step, the pre-charging method achieved a 10–12% reduction in 10–90% settling time relative to the grounded-input baseline.
- For a large voltage step, the pre-charging circuit provided a ~23% reduction in time to reach within ±10% of the final value.

These improvements were achieved without the need to slow down the sampling sequence or sacrifice valid measurements, as is required with the floating-input approach. Moreover, the pre-charged transitions showed lower overshoot and more controlled exponential settling behavior, contributing to increased accuracy and stability in high-impedance sensor interfacing.

The grounded-input method, while simple and deterministic, consistently resulted in longer settling times due to the repeated need to charge the ADC node from 0 V. The floating-input method delayed transients but at the cost of throughput and predictability, since it relies on passive decay and introduces variability based on prior states and parasitics.

In contrast, the pre-charging approach directly addresses the source of charge redistribution by initializing the ADC input node to a level near half of the ADC reference. This active preparation reduces the load on high-impedance sources and enables quicker stabilization, making it especially valuable in energy-constrained or time-sensitive applications such as wireless sensor nodes, energy-harvesting systems, and multiplexed analog front-ends.

These results confirm the pre-charging scheme as feasible in its discrete form; however, practicality and effectiveness could be further improved.

**Figure 23:**
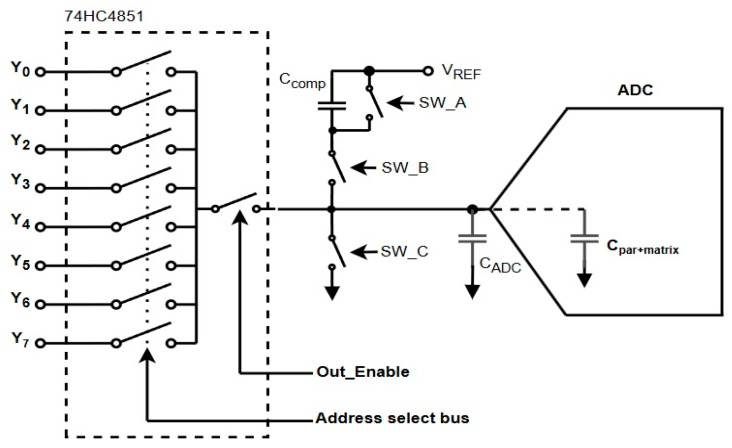
Pre-charging scheme diagram with external capacitor at ADC input.

Since the transient behavior of C_ADC_ and C_COMP_ results in values much larger than the typical input capacitance of most ADCs (a few picofarads to tens of picofarads), an external capacitor C_ADC_ is also needed based on the current selection of switching transistors (BSS84—PMOS and BS170—NMOS) (Figure 23). An optimal value for our experimental setup was determined to be in the region of 2.2 nF to 22 nF. Higher values are seen to mitigate charge injection effects almost completely at the expense of longer settling times and larger voltage dips in the used reference voltage line, which in our proposal, could be the main supply regulator in order to avoid loading on the internal ADC reference.

### 3.4. Used Equipment

Measurement equipment:

The Analog Discovery v1 was used with software version v.3.20.1.

- Two channel oscilloscopes (1 MΩ, ±25 V, differential, 14 bit, 100 MS/s.
- Two channel arbitrary function generator (22Ω, ±5 V, 14 bit, 100 MS/s.
- Sixteen-channel digital logic analyzer (CMOS, 100 MS/s)—5 V tolerant.

Oscilloscope probes:

- UNI-T, model UT-P03 with 85 to 115 pF input capacitance in the ×1 configuration and 14.5 to 17.5 pF in the ×10.

Power supply:

- ATTEN TPR75-2A

Microcontroller board:

- STM32 Nucleo F411RE and Arduino Uno R3

## 4. Results Summary and Discussion

This work presented the grounds for a low component count discrete capacitive pre-charging circuit for improving the transient response and settling performance of ADC inputs in multiplexed embedded systems. By actively conditioning the ADC input node to an intermediate voltage before switching channels, the proposed method effectively mitigates charge redistribution effects and reduces the time required for accurate signal acquisition. Compared with conventional approaches—grounding or floating unused multiplexer inputs—the pre-charging scheme consistently demonstrated superior performance, achieving up to 23% faster settling in large voltage transitions with minimal hardware complexity.

The results validate the approach as a practical enhancement for sensor interfaces, particularly in energy-constrained and high-impedance applications such as wireless sensor nodes, energy-harvesting systems, and IoT platforms. Its implementation relies on widely available components and requires no specialized analog circuitry, making it highly accessible for cost-sensitive designs. Moreover, the technique aligns with broader trends in low-power embedded system design, where digital coordination increasingly complements analog signal conditioning.

### Future Work

While future work may explore adaptive pre-charge levels, integration into programmable logic, or scaling to higher channel-count architectures, the present study establishes a clear foundation for enhancing ADC interfacing through simple and effective preconditioning.

Here are the noted open points for further improvement and analysis:

- Definition of Figure of Merit (FoM) applicable to the proposed pre-charging scheme, allowing direct similar to the FoMs commonly used.
- Measurement and analysis of Kickback magnitude at multiplexor signal sources.
- Further measurements on ADC accuracy in terms of slewing related gain error and dynamic performance parameters such as SPFDR, THDN, and ENOB.
- Estimation of long-term stability caused by used component parameter drift and environment conditions, such as aging and thermal noise in the used capacitors.
- Experiments with lower total gate capacitance transistors and dynamic voltage scaling.

Due to flaws in the designed schematic and PCB, not all multiplexer address transitions could be evaluated and compared directly. This issue needs to be addressed with an improved version of the test setup.

Further reducing the size of the needed CCOMP and CADC is required to make this approach more feasible. In its above evaluated form, the bulk of consumed current is drawn from the VREF line, which was the already available supply regulator and not the ADC internal reference. Since both capacitors are of equal value (e.g., 2.2 nF) and connected in series, the energy required per every sample could be written as follows:

(6) $$E_{Sample}\;=\;\frac{C_{equivalent}{\times \;V}_{REF}^{2}}{2}\;=\;\frac{1.1nF\times 25V}{2}=13,7nJ/sample$$

If a sampling rate of 100kSmps is required, this would result in an average consumption of 1.375 mW for the pre-charging scheme alone. This can be compared to the use of operational amplifiers for ADC matrix drivers at the same sampling rate shown in the Table 2. Current consumption values for comparing the results were taken from a paper on maximizing power savings in SAR ADC systems from 2018 [45].

## Figures and Tables

**Table 1:** Proposed truth table of the control lines for the pre-charging circuit.

	HI-Z	Reset Phase	Break-Before-Make	Pre-Charging Phase
SW_A	“1”	“0”	“1”	“1”
SW_B	“1”	“1”	“1”	“0”
SW_C	“0”	“1”	“0”	“0”
EN_N	“0”	“1”	“1”	“1”
BSRR equiv.	0 × 3	0 × E	0 × B	0 × 9
Duration	1.0 × 10^−6^ s	4.0 × 10^−7^ s	2.0 × 10^−8^ s	2.0 × 10^−8^ s
Num. clocks	40	16	1	1

**Table 2:** Comparison of power consumption for the proposed pre-charging scheme and selected operational amplifiers at various sampling rates.

Amplifier	Sample Rate	Power Consumption	This Work (C_ADC_, C_COMP_ = 2.2 nF)
LPV811	1 kSps	1.8 µW	13.75 µW
TLV313	100 kSps	192.7 µW	1375 µW
OPA320	1 MSps	6386 µW	13.75 mW

## Data Availability

Data are contained within the article and Supplementary Materials.

## Supplementary Materials

Uploaded on github with a public license https://github.com/bshabanski/stm32_bsrr_mux_control (accessed on 5 August 2025) [43].
